# Juvenile Galápagos Pelicans Increase Their Foraging Success by Copying Adult Behaviour

**DOI:** 10.1371/journal.pone.0051881

**Published:** 2012-12-14

**Authors:** Henrik Brumm, Irmgard Teschke

**Affiliations:** 1 Max Planck Institute for Ornithology, Communication and Social Behaviour Group, Seewiesen, Germany; 2 Max Planck Institute for Ornithology, Sensory Ecology Group, Seewiesen, Germany; Università di Parma, Italy

## Abstract

Social learning is the building block of culture and traditions in humans and nonhuman animals, and its study has a long history. Most investigations have addressed either the causation or the function of social learning. Though much is known about the underlying mechanisms of social learning, demonstrations of its adaptive value in a natural setting are lacking. Here we show that juvenile brown pelicans (*Pelecanus occidentalis*) can increase their foraging efficiency by copying adult diving behaviour, suggesting that social learning helps juveniles to find profitable food patches. Our findings demonstrate the potential fitness consequences of behavioural copying and thus highlight the possible adaptive importance of social learning.

## Introduction

Social learning has fascinated sociologists, psychologists and biologists for a long time, and the last two decades have seen an explosion of both theoretical and empirical studies on social learning in animals [1], [2], [3], [4]. In social learning, animals change their behaviour based upon information transferred from one individual to another. A prime example of social learning in animals is vocal learning [5] and vocal production learning in birds has been extensively studied [6], [7], [8]. Social transmission of behaviour can lead to copying [9], in which one animal matches the behaviour of another, thereby reproducing, for instance, patterns of movement or patch choices [10], [11], [12]. Behavioural copying is employed by many species to acquire foraging information [13], [14], [15], [16]. Naïve fish, for instance, can learn the route to a food source by swimming with informed conspecifics, and in the process they copy the route from the other fish [17].

In the case of strongly heritable, species-typical foraging behaviour, vertical genetic transmission between generations takes place reliably and with high fidelity but on the other hand, such behaviour is also potentially less flexible in fluctuating environments. Various mathematical models have been developed to investigate the emergence of social learning in relation to non-social learning as well as the potential fitness consequences of social learning [18], [19], [20], [21]. Another line of research attempts to link evidence for social learning with its value in the real world [reviewed in 13, 22, 23]. As far as foraging behaviour is concerned, such studies range from those which show the benefits of social learning in experimental situations in captivity that are designed to mimic real-world foraging problems, e.g. [24], [25], [26], [27] to studies conducted in the wild with field experiments that have been seeded by an experimenter, e.g. [15], [28], [29], [30], [31]. However, only few studies have demonstrated naturally occurring social learning in foraging animals in the wild. One notable exception is the work by Thornton & McAuliffe [32] who showed that adult meerkats provide pups with opportunities to practice skills of handling live prey, thus facilitating learning. In addition to the scarcity of field studies on naturally occurring social learning, previous research on foraging abilities has primarily looked at the acquisition of certain skills, such as solving a special task or handling a particular food item, e.g. [33], [34], [35]. However, to link social learning to survival and reproductive success, it is crucial to show that social learning improves vital foraging skills or foraging performance in general. Such a demonstration is imperative to a fuller understanding of the benefits of social learning in the wild, and, ultimately, may help elucidate the reasons that social learning has evolved in the first place.

**Figure 1:**
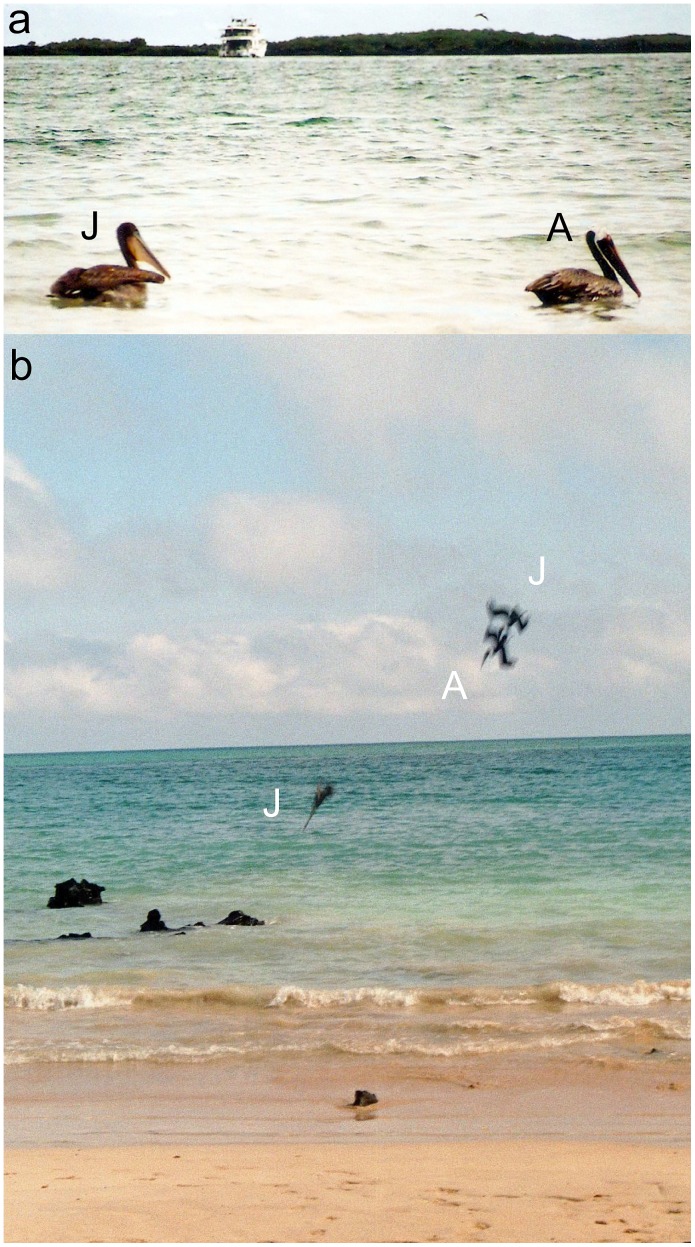
Brown pelicans (*Pelicanus occidentalis*) in the Galápagos Islands. **a**, age classes can easily be distinguished by their plumage: juveniles (J) have dark-brown heads and necks whereas adults (A) have white heads. **b**, plunge diving pelicans hunting for fish: a solo diving juvenile (centre) and a juvenile following an adult (right).

Here we present observational data on the social effects on the general foraging efficiency in wild brown pelicans (*Pelecanus occidentalis*). In particular, we show the value of the presence of an experienced demonstrator for juveniles which must master the species-typical foraging technique known as plunge diving. In many animals, the juvenile phase is a critical period both in terms of survival and social learning [8], [36], [37], [38]. Juvenile brown pelicans (Figure 1a) have a lower foraging efficiency than adults [39], [40], [41] and as a result many juveniles die of starvation [42]. Thus, the rapid acquisition of hunting skills in this species is a crucial process and its outcome has decisive fitness consequences.

The brown pelican is the only pelican species that dives for fish: foraging birds fly slowly at about 9 m above the water surface and upon sighting prey, suddenly check their flights and dive bill first into the water [43]. This so-called plunge diving (Figure 1b) is a challenging task because in addition to the evasive tactics of the prey, diving pelicans must contend with other obstacles such as surface glare, refraction, and wind. The inferior foraging performance of juveniles hints at a deficit in the motor skills necessary for diving and catching fish or a lack of experience in selecting profitable food patches or both [44]. Since the juvenile period is a critical ontogenetic bottleneck because of the starvation risk [42] it is crucial for the survival of young pelicans to rapidly develop efficient hunting techniques in the transition to adulthood. Here we provide observational evidence suggesting that social learning may play a decisive role in the improvement of the general foraging efficiency of immature birds.

## Methods

We studied the hunting behaviour of brown pelicans in the Galápagos Archipelago in January 2008 and February 2009. Mixed groups of adults and juveniles were observed at six different locations in the coastal waters of the islands of Santa Cruz, Baltra, and Isabella. The distances between the observing locations ranged between 20 and 96 km. Pelican group sizes varied between 3 and 17 birds with a mean number of 3.2 adults and 4.3 juveniles per group (ranges: 2–4 adults and 1–13 juveniles). Age classes were determined by plumage. Brown pelicans commonly acquire adult plumage around two years of age. Birds having dark-brown heads and necks were classified as juvenile, birds with white heads and necks were classed as adults [39], [40]. The observed groups included all individuals that foraged within eyesight range at a given location. In all six cases, the groups remained constant during the entire observation period, i.e. no bird left the location and no new bird joined the group.

All foraging was confined to within 30 meters of the islands, and could be observed from shore without the aid of binoculars. The hunting behaviour of the pelicans at each location was continuously observed and all plunge dives and their outcome were recorded in a field notebook. At each location, all observations were made during one day and each location was only visited once. Three locations were visited in the morning between 0600 and 1100 hours and three sites in the afternoon between 1400 and 1700 hours. Average observation time was 86 minutes per location (range: 35–120 minutes). In total, 532 diving events were observed (151 dives by adults, 310 by juveniles, 71 by juveniles copying an adult).

The success of pelican plunge dives is easy to score because a toss of the head is indicative of swallowing [39], [43]. If no catch is made, birds pull their heads out of the water with the bill open, allowing the water to drain out immediately, and the head and bill rapidly return to the normal position. Successful birds, on the other hand, drain the water from their bill slowly, and then swallow their prey by tossing the head backwards.

Before diving, foraging birds fly slowly above the water surface at a height of about 9 m, looking for prey [45]. Usually, the search flights of different birds do not seem to be spatially coordinated but sometimes a juvenile may closely follow a hunting adult (usually within a few meters). When the adult is diving, the following juvenile will dive immediately after it, so that its bill will enter the water surface within 1 or 2 meters from the preceding adult. Based on prior observations, we used an operational criterion of diving within 1 second after the adult. At five of the six study sites, only four or fewer juveniles were observed and all of these dived on their own as well as with an adult.

We investigated differences in foraging success between adults, juveniles, and juveniles following adults with generalized linear mixed-effects models (GLMMs) calculated in R 2.10.1 (R Development Core Team 2009). As we could not distinguish individuals in all cases, we included site as a random effect rather than individual. Thus, the overall sample size was N = 6 study sites. The GLMMs were fitted with a binomial error structure, using the function lmer (R package lme4). We used a model comparison approach (Wald χ^2^ test) to assess whether omitting the factor “group” from the model caused a significant change of the model fit. As post hoc tests, we quantified the strength of evidence for differences between classes of pelicans with simultaneous tests for General Linear Hypotheses, using the multcomp package in R [46].

### Ethical Statement

This field study consisted solely of observations from publicly accessible sites of the Galapagos National Park. Both researchers had permits to enter the national park. Animals were not manipulated or otherwise disturbed through these observations. Therefore, no special permit was required for this study.

## Results

**Figure 2:**
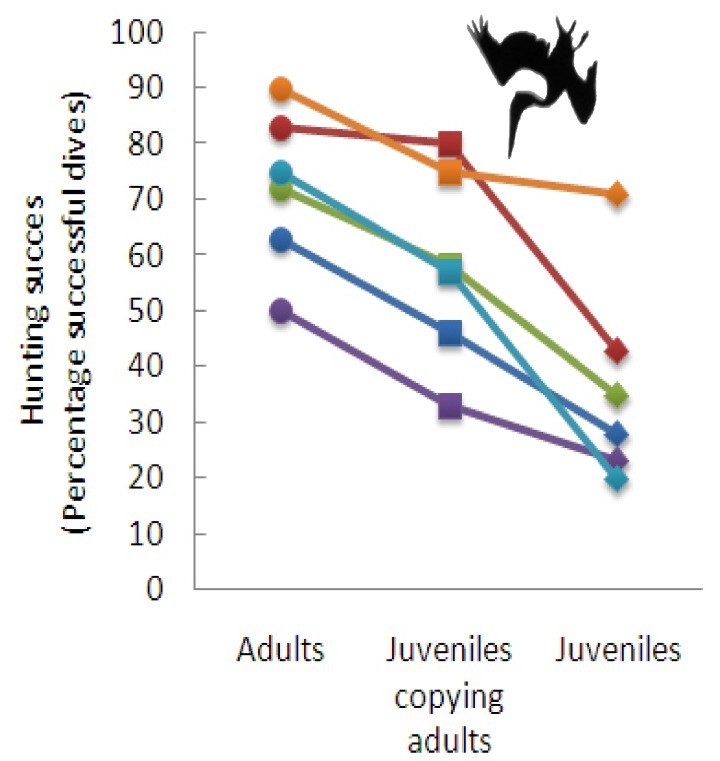
Foraging success of adult and juvenile brown pelicans. Data are shown for all six study sites.

We found that the pelican classes (adults, juveniles, and juveniles following adults) differed markedly in their hunting efficiency (Wald test: *χ^2^* = 63.3, *df* = 2, *p*<0.0001, see Figure 2), with juveniles having a lower hunting efficiency than adults (estimate: −1.6930, SE: 0.2261, *z* = −7.487 *p*<0.001). However, we also discovered that juvenile birds sometimes copied adult fishing behaviour. In these instances, juveniles followed a hunting adult closely, immediately following adult dives and plunging into the water within 1 or 2 meters of the same spot (Figure 1b). Such behavioural matching was frequently observed, with juveniles following an adult in 23% of all their dives. However, we never observed that an adult bird copied the diving behaviour of another adult nor that a juvenile copied another juvenile.

There was strong evidence that copying adult behaviour increases the foraging success of the juvenile pelicans (estimate: −0.9765, SE: 0.2792, *z* = −3.498, *p* = 0.001, see Figure 2): When diving on their own, juveniles caught fish on an average of 33% (95% CI = 0.22–0.46) of all hunting attempts (estimated average success rate as estimated from the model), but they raised their proportion of successful plunge dives to 57% (95% CI = 0.39–0.73) when reproducing the adult fishing behaviour. However, even the increased hunting success of socially foraging juveniles was below adult performance (estimate: −0.7165, SE: 0.3018, *z* = −2.374, *p* = 0.045, see Figure 2). The foraging success of the adults was not affected by the presence of a juvenile (Wald test: *χ^2^* = 0.57, *df* = 1, *p* = 0.449).

## Discussion

We observed that juvenile brown pelicans substantially increased their hunting success when copying adult diving behaviour. This increase in juvenile foraging efficiency in the presence of an adult suggests that the poorer hunting success in juvenile birds is not entirely attributable to a lack of the motor skills necessary for capturing fish. Instead, immature pelicans may be less capable of correctly evaluating the profitability of foraging patches, e.g. by assessing prey density. By copying the patch choice of adults, juveniles benefit from the adults’ knowledge to recognize profitable feeding opportunities. It is most likely that this gain arises through the behavioural synchrony which leads to diving in the same spot. Knowledge transfer probably occurs in a second step, when the matching of adult behaviour enables juvenile birds to eventually learn to distinguish profitable food patches from unprofitable ones on their own. Both steps together would result in learning that is functionally equivalent to contextual imitation, where an observer learns to use an existing action in a novel context [47]. However, our data also show that even the increased foraging success of copying juveniles was below adult performance, which may reflect an age-related difference in motor skills. Thus, immature pelicans may increase their hunting abilities in two independent ways: profitable patch choice may be enhanced by social learning, whereas individual learning may be needed to improve physical foraging skills.

Alternative interpretations of our observation that do not require social learning may involve the notion that the probability of following a diving adult could be affected by the hunting skills of juveniles. For example, older juvenile pelicans might be both more likely to follow adults and more likely to successfully capture fish than younger juvenile pelicans, or juvenile pelicans that are more adept at catching fish might be more likely to follow adults. However, we are confident that these alternative hypotheses cannot explain our findings. At five of our six study sites, only a small number of juveniles were present (4 or fewer), enabling individual identification throughout the observation period, and all of these dived on their own as well as with an adult. This means that the overall effect of increased hunting success of copying birds cannot be attributed to different individuals engaging in social and individual foraging.

Many animals travel to and feed in locations where they can see conspecifics feeding [13]. This is an example of one particular mechanism of social information acquisition known as ‘local enhancement’ [48]. This process is important in finding and learning about foraging sites in many taxa, including birds, fish, mammals, and insects [13], [49]. Local enhancement may also account for the observation that juvenile marsh harriers (*Circus aeruginosus*) increased their foraging success when they hunted in the same area together with adult birds [50]. The young harriers caught more prey when foraging not further away than 50 meters from an adult but, unlike in our study, the juvenile birds did not match the movements of the adult birds. Thus, it seems that juvenile harriers may be attracted by local enhancement to food patches where adults hunt but then they forage independently once they arrive there. A similar process of local enhancement and subsequent independent foraging has also been suggested for feeding great tit (*Parus major*) flocks [51]. However, local enhancement cannot account for the phenomenon reported here, since we did not observe that juvenile pelicans gravitated towards the location where they observed foraging adults per se, rather they closely followed an adult during flight and meticulously matched its diving behaviour. In other words, the foraging patch choices of the juveniles could not have been the result of their increased attention to the location where an adult dived because of the very short time lag of less than a second between the patch choice of the adult and the matched choice of the juvenile. Figure 1b illustrates how closely the observer foraging matches those of the demonstrator, both in terms of timing and location.

Other cases of patch choice copying that show similar matching in time and space were reported in laboratory studies with foraging fishes [52]. In particular, fish have been shown to follow informed individuals to find food patches [17]. Complex underlying social learning mechanisms need not be invoked in order to explain such guided social learning. Path copying in fish or patch choice copying in plunge diving pelicans could be explained given certain prerequisites like the motivation to shoal in the example of the fish or in the case of the pelicans, social tolerance between a demonstrator and observer, along with the motivation of observers to replicate specific demonstrator motor patterns (contagion) and the ultimate attainment of a food reward upon accurate copying of behaviour [53]. Further work would be needed to elucidate the exact mechanisms involved in the development of pelican plunge diving behaviour.

To support our interpretation of the data, a crucial next step would be to follow individual juvenile pelicans over time to see whether there is an improvement in solo diving skill that is related to the degree of their previous association with diving adults. Regarding the transfer of socially acquired information between individuals it would furthermore be interesting to know from whom juvenile pelicans copy. The cases in which we could distinguish individual birds suggested that each juvenile only followed one or two particular adult birds, raising the possibility that young birds may learn from their parents. However, it is rather unlikely that the reported social learning of hunting behaviour in immature pelicans involves teaching [54], for we did not observe any signs of active facilitation of learning by the demonstrating adults (e.g. by slowing down their flight to ensure that the juveniles could stay in close proximity). Also, the adults did not suffer a reduction in foraging success because of the juvenile’s presence.

The increased foraging success of copying juveniles in comparison to non-copying birds clearly indicates the potential fitness consequences of social learning. Beyond that, our findings highlight the adaptive importance of copying others, since behavioural matching considerably improved individual hunting success in brown pelicans, which, in turn, will increase the probabilities of survival and reproduction.

## References

[1] Laland KN, Galef BG (2009) The Question of Animal Culture. Cambridge: Harvard University Press.

[2] Tomasello M, Kruger AC, Ratner HH (1993) Cultural learning. Behavioral and Brain Sciences 16: 495–511.

[3] Danchin E, Giraldeau LA, Valone TJ, Wagner RH (2004) Public information: From nosy neighbors to cultural evolution. Science 305: 487–491. 15273386 10.1126/science.1098254

[4] Heyes CM (1994) Social learning in animals: Categories and mechanisms. Biological Reviews of the Cambridge Philosophical Society 69: 207–231. 8054445 10.1111/j.1469-185x.1994.tb01506.x

[5] Janik VM, Slater PJB (2000) The different roles of social learning in vocal communication. Anim Behav 60: 1–11. 10924198 10.1006/anbe.2000.1410

[6] Beecher MD, Brenowitz EA (2005) Functional aspects of song learning in songbirds. Trends in Ecology & Evolution 20: 143–149. 16701358 10.1016/j.tree.2005.01.004

[7] Catchpole CK, Slater PJB (2008) Bird song. Biological Themes and Variations. Cambridge: Cambridge University Press.

[8] Hultsch H, Todt D (2008) Comparative aspects of song learning. In: Zeigler HP, Marler P, editors. Neuroscience of Birdsong. Cambridge: Cambridge University Press. 204–216.

[9] Galef BG (1988) Imitation in animals: History, definition, and interpretation of data from the psychological laboratory. In: Zentall TR, Galef BG, editors. Social Learning: Psychological and Biological Perspectives. Hillsdale, New Jersey: Lawrence Erlbaum. 3–28.

[10] Galef BG, Laland KN (2005) Social learning in animals: Empirical studies and theoretical models. Bioscience 55: 489–499.

[11] Leadbeater E, Chittka L (2007) Social learning in insects - from miniature brains to consensus building. Current Biology 17: R703–R713. 17714664 10.1016/j.cub.2007.06.012

[12] Sumpter DJT, Krause J, James R, Couzin ID, Ward AJW (2008) Consensus decision making by fish. Current Biology 18: 1773–1777. 19013067 10.1016/j.cub.2008.09.064

[13] Galef BG, Giraldeau LA (2001) Social influences on foraging in vertebrates: causal mechanisms and adaptive functions. Animal Behaviour 61: 3–15. 11170692 10.1006/anbe.2000.1557

[14] Rapaport LG, Brown GR (2008) Social influences on foraging behavior in young nonhuman primates: Learning what, where, and how to eat. Evolutionary Anthropology 17: 189–201.

[15] Müller CA, Cant MA (2010) Imitation and traditions in wild banded mongooses. Current Biology 20: 1171–1175. 20605459 10.1016/j.cub.2010.04.037

[16] Pike TW, Laland KN (2012) Conformist learning in nine-spined sticklebacks' foraging decisions. Biology Letters 6: 466–468. 10.1098/rsbl.2009.1014 PMC2936200 20129948

[17] Laland KN, Williams K (1997) Shoaling generates social learning of foraging information in guppies. Animal Behaviour 53: 1161–1169. 9236013 10.1006/anbe.1996.0318

[18] Boyd R, Richerson PJ (1985) Culture and the Evolutionary Process. Chicago: University of Chicago Press.

[19] Kendal J, Giraldeau LA, Laland K (2009) The evolution of social learning rules: Payoff-biased and frequency-dependent biased transmission. Journal of Theoretical Biology 260: 210–219. 19501102 10.1016/j.jtbi.2009.05.029

[20] Borenstein E, Feldman MW, Aoki K (2008) Evolution of learning in fluctuating environments: When selection favors both social and exploratory individual learning. Evolution 62: 586–602. 18182074 10.1111/j.1558-5646.2007.00313.x

[21] Enquist M, Eriksson K, Ghirlanda S (2007) Critical social learning: A solution to Rogers's paradox of nonadaptive culture. American Anthropologist 109: 727–734.

[22] Reader S, Biro D (2010) Experimental identification of social learning in wild animals. Learning & Behavior 38: 265–283. 20628165 10.3758/LB.38.3.265

[23] Thornton A, Clutton-Brock T (2011) Social learning and the development of individual and group behaviour in mammal societies. Philosophical Transactions of the Royal Society B: Biological Sciences 366: 978–987. 10.1098/rstb.2010.0312 PMC3049086 21357220

[24] Page RA, Ryan MJ (2006) Social transmission of novel foraging behavior in bats: frog calls and their referents. Current Biology 16: 1201–1205. 16782010 10.1016/j.cub.2006.04.038

[25] Weigl PD, Hanson EV (1980) Observational learning and the feeding behavior of the red squirrel *Tamiasciurus hudsonicus*: The ontogeny of optimization. Ecology 61: 214–218.

[26] Tebbich S, Taborsky M, Fessl B, Blomqvist D (2001) Do woodpecker finches acquire tool-use by social learning? Proceedings of the Royal Society of London B 268: 2189–2193. 10.1098/rspb.2001.1738 PMC1088865 11674865

[27] Aisner R, Terkel J (1992) Ontogeny of pine cone opening behaviour in the black rat, *Rattus rattus* . Animal Behaviour 44, Part 2: 327–336.

[28] Midford PE, Hailman JP, Woolfenden GE (2000) Social learning of a novel foraging patch in families of free-living Florida scrub-jays. Animal Behaviour 59: 1199–1207. 10877899 10.1006/anbe.1999.1419

[29] Langen TA (1996) Social learning of a novel foraging skill by white-throated magpie-jays (*Calocitta formosa*, Corvidae): A field experiment. Ethology 102: 157–166.

[30] Slagsvold T, Wiebe KL (2011) Social learning in birds and its role in shaping a foraging niche. Philosophical Transactions of the Royal Society B: Biological Sciences 366: 969–977. 10.1098/rstb.2010.0343 PMC3049099 21357219

[31] Bouchard J, Goodyer W, Lefebvre L (2007) Social learning and innovation are positively correlated in pigeons (*Columba livia*). Animal Cognition 10: 259–266. 17205290 10.1007/s10071-006-0064-1

[32] Thornton A, McAuliffe K (2006) Teaching in wild meerkats. Science 313: 227–229. 16840701 10.1126/science.1128727

[33] Holzhaider JC, Hunt GR, Gray RD (2010) Social learning in New Caledonian crows. Learning & Behavior 38: 206–219. 20628160 10.3758/LB.38.3.206

[34] Boogert NJ, Reader SM, Hoppitt W, Laland KN (2008) The origin and spread of innovations in starlings. Animal Behaviour 75: 1509–1518.

[35] Lefebvre L (1986) Cultural diffusion of a novel food-finding behavior in urban pigeons - an experimental field-test. Ethology 71: 295–304.

[36] Magrath RD (1991) Nestling weight and juvenile survival in the blackbird, *Turdus merula* . Journal of Animal Ecology 60: 335–351.

[37] Gaillard JM, Festa-Bianchet M, Yoccoz NG, Loison A, Toigo C (2000) Temporal variation in fitness components and population dynamics of large herbivores. Annual Review of Ecology and Systematics 31: 367–393.

[38] Brown C, Laland K (2001) Social learning and life skills training for hatchery reared fish. Journal of Fish Biology 59: 471–493.

[39] Orians GH (1969) Age and hunting success in brown pelicans (*Pelecanus occidentalis*). Animal Behaviour 17: 316–319.

[40] Brandt CA (1984) Age and hunting success in the brown pelican: influcens of skill and patch choice on foraging efficiency. Oecologia 62: 132–137. 28310751 10.1007/BF00377386

[41] Arnqvist G (1992) Brown pelican foraging success related to age and height of dive. Condor 94: 521–522.

[42] Jackson MH (1993) Galapagos: A Natural History. Calgary: University of Calgary Press.

[43] Schreiber RW, Woolfenden GE, Curtsinger WE (1975) Prey capture by brown pelicans. Auk 92: 649–654.

[44] Coblentz BE (1986) A possible reason for age-differential foraging success in brown pelicans. Journal of Field Ornithology 57: 62–63.

[45] Carl RA (1987) Age-class variation in foraging techniques by brown pelicans. Condor 89: 525–533.

[46] Hothorn T, Bretz F, Westfall P (2008) Simultaneous inference in general parametric models. Biometrical Journal 50: 346–363. 18481363 10.1002/bimj.200810425

[47] Byrne RW (2002) Imitation of novel complex actions: What does the evidence from animals mean? Advances in the Study of Behavior 31: 77–105.

[48] Thorpe WH (1963) Learning and instinct in animals. Cambridge, MA: Harvard University Press.

[49] Hoppitt W, Laland KN (2008) Social processes influencing learning in animals: A review of the evidence. Advances in the Study of Behavior 38: 105–165.

[50] Kitowski I (2009) Social learning of hunting skills in juvenile marsh harriers *Circus aeruginosus* . Journal of Ethology 27: 327–332.

[51] Krebs JR, MacRoberts MH, Cullen JM (1972) Flocking and feeding in the great tit *Parus major* - an experimental study. Ibis 114: 507–530.

[52] Laland KN, Atton N, Webster MM (2011) From fish to fashion: experimental and theoretical insights into the evolution of culture. Philosophical Transactions of the Royal Society B-Biological Sciences 366: 958–968. 10.1098/rstb.2010.0328 PMC3049094 21357218

[53] Zentall TR (2012) Perspectives on observational learning in animals. Journal of Comparative Psychology 126: 114–128. 21895354 10.1037/a0025381

[54] Hoppitt WJE, Brown GR, Kendal R, Rendell L, Thornton A, et al (2008) Lessons from animal teaching. Trends in Ecology & Evolution 23: 486–493. 18657877 10.1016/j.tree.2008.05.008

